# The effect of topical antibiotic or antibiotic-corticosteroid treatment on the ocular surface microbiota of healthy horses

**DOI:** 10.3389/fmicb.2025.1535095

**Published:** 2025-08-04

**Authors:** M. G. Martin de Bustamante, C. E. Plummer, B. Caddey, D. E. Gomez

**Affiliations:** ^1^Department of Small Animal Clinical Sciences, College of Veterinary Medicine, University of Florida, Gainesville, FL, United States; ^2^Faculty of Veterinary Medicine, University of Calgary, Calgary, AB, Canada; ^3^Department of Internal Medicine, Abu Dhabi Equine and Camel Hospital (ADECH), Abu Dhabi, United Arab Emirates

**Keywords:** antimicrobial, bacteria, conjunctiva, equine, microbiome, dexamethasone

## Abstract

**Introduction:**

Information regarding the impact of topical antibiotics with or without corticosteroids on the microbiota of the horses’ eyes is limited. This study aimed to describe the bacterial ocular surface microbiota in healthy horses and evaluate the effect of topical antibiotics or antibiotic-corticosteroid medication on the ocular surface microbiota.

**Methods:**

This was a prospective, randomized, longitudinal, blinded study in which one eye of 12 horses was treated 3 times daily for 7 days with neomycin, polymyxin B and bacitracin ophthalmic ointment (*n* = 6) or neomycin, polymyxin B and dexamethasone ophthalmic ointment (*n* = 6). The contralateral eyes operated as untreated controls. The inferior conjunctival fornix of both eyes was sampled at baseline before antibiotic administration (day 0), on days 3, 7, 9, 14, and 30. The ocular surface microbiota was characterized by amplifying the V4 region of the 16S ribosomal RNA gene.

**Results:**

Alpha- (richness and diversity) and beta-diversity (weighted and unweighted UniFrac distances) measurements of the ocular surface microbiota varied similarly after treatments starting on day 1, returning to baseline measurements by day 30. At baseline, the main phyla detected in the ocular microbiota was Proteobacteria, representing 75% relative abundance, followed by Firmicutes and Bacteroidetes. After treatments, Proteobacteria declined in all groups, and Firmicutes and Bacteroidete’s relative abundance increased, returning to baseline levels on day 30. The main genera detected on the ocular surface on day 0 were *Suttonella*, *Nicoletella, Pasteurella*, and members of the family Moraxellaceae. After treatment, the relative abundance of this bacteria declined in all groups, returning to baseline levels on day 30, although some alterations were still present.

**Discussion:**

Here we show that topical antibiotics administered with or without corticosteroids induce changes in the ocular surface of horses’ eyes, and the microbiota appears to return to baseline approximately three weeks after treatment discontinuation.

## 1 Introduction

The ocular surface consists of the tear film and the conjunctival and corneal epithelia. It serves as the interface between the globe and the external environment, providing an essential first line of defense against pathogenic microorganisms (Weese and Gomez-Nieto, 2016). This protective function is accomplished by a combination of several mechanisms, including a physical barrier (e.g., intact epithelium), antimicrobial compounds present in tears (e.g., lysozyme, lactoferrin, ß-lysin, and defensins), and immune-mediated defenses (e.g., complement and IgA) (McDermott, 2013). Despite these protective features, in health, the ocular surface remains colonized by a resident population of microorganisms (the ocular surface microbiota), albeit in much lower numbers than at other sites such as the gastrointestinal tract (Ozkan and Willcox, 2019).

Using traditional culture-dependent techniques, the resident bacterial composition of the equine ocular surface has been well-described previously, both in healthy horses and horses with ulcerative keratitis (Moore et al., 1983, 1995; Whitley and Moore, 1984; Sauer et al., 2003; Keller and Hendrix, 2005; de Sousa et al., 2011; Johns et al., 2011). The cultured bacteria varies with age, sex, housing, and climate (Gomez and Weese, 2017). The proportion of bacterial communities on the ocular surface changes with disease, specifically ulcerative keratitis and topical antibiotic use (Moore et al., 1983, 1995). Culture-dependent techniques have reported various positive cultures in normal horses ranging from 30 to 100%, depending on the specific study (Whitley and Moore, 1984; Gemensky-Metzler et al., 2005; Johns et al., 2011). This variability in the detection of bacteria on the ocular surface using culture is not necessarily a reflection of sterility; rather, it is likely reflective of the limitations of culture-based techniques. Recently, culture-independent techniques have been utilized to characterize further the ocular surface microbiota in humans and several veterinary species, including dogs, cats, horses, koalas, and finches (Weese et al., 2015; Thomason et al., 2017a, 2017b; Vidgen et al., 2017; Leis and Costa, 2019; Scott et al., 2019; LaFrentz et al., 2020; Walsh et al., 2021; Santibáñez et al., 2022). These studies have revealed a much richer and more diverse microbial community than previously believed.

Topical ophthalmic antibiotics or antibiotic-corticosteroid medications are frequently used to treat some horses’ most common ophthalmic diseases, such as ulcerative keratitis and equine recurrent uveitis, respectively (Keller and Hendrix, 2005; Gerding and Gilger, 2016). Numerous and diverse organisms have been reported to infect the equine cornea. In most cases, infectious keratitis results from opportunistic colonization of exposed corneal stroma by commensal microbiota. Staphylococcus, Streptococcus, and Pseudomonas species are the most isolated pathogens from infected corneal wounds using traditional culture techniques (Keller and Hendrix, 2005; Gerding and Gilger, 2016). The most commonly used antibiotics include neomycin (gram-negative spectrum), polymyxin B (gram-positive spectrum) and bacitracin (gram-positive spectrum). Contradictory results have been published regarding the effect of antibiotics on ocular surface microbiota, with studies showing a shift from predominantly gram-positive to gram-negative organisms and no alteration in the main taxa after antibiotic administration (Whitley and Moore, 1984; Andrew et al., 1998; Gemensky-Metzler et al., 2005). Topical corticosteroids can inhibit corneal epithelial wound healing, potentiate corneal stromal degradation, and impair phagocyte response and neovascularization (Brown et al., 1970; Petroutsos et al., 1982; Boneham and Collin, 1995; Gemensky-Metzler et al., 2005). Consequently, these alterations in local immunity and corneal repair mechanisms can increase susceptibility to infectious diseases of the eye. Although information regarding the balanced bacterial communities in the ocular surface and the effect of antibiotics and corticosteroids is available, mainly using culture-based methodologies, limited reports in the literature characterizing the normal bacterial ocular surface microbiota in horses using high throughput techniques or examining the effects of topical ophthalmic medications on this microbial population are available. This study aims to describe the bacterial ocular surface microbiota in healthy horses and to evaluate the effect of topical antibiotics or antibiotic-corticosteroid medication on the ocular surface microbiota. We hypothesized that a stable conjunctival microbiota would be present over the sampling period with limited effect from topical antibiotics or antibiotic-corticosteroid administration.

## 2 Materials and methods

### 2.1 Animals and tissues used

This study was performed following the guidelines of the Institutional Animal Care and Use Committee of the University of Florida (IACUC Study #201810419). Twelve healthy horses from the University of Florida, College of Veterinary Medicine Equine Research Program (ERP) Shared Herd received complete ophthalmic examinations with standing sedation by a board-certified veterinary ophthalmologist and ophthalmology resident at the University of Florida Large Animal Hospital (UF-LAH). Following sedation with detomidine (0.015 mg/kg IV) ± xylazine (0.2–0.3 mg/kg IV PRN at clinician discretion), both eyes were dilated with topical administration of 0.1 mL tropicamide ophthalmic solution (1%). An auriculopalpebral motor block was performed in both eyes with 2 mL of lidocaine hydrochloride solution (2%) to facilitate examination. All horses received a complete ophthalmic examination, including anterior segment evaluation (Kowa SL-15; Kowa Company Ltd.) and posterior segment examination with indirect ophthalmoscopy (Keeler Vantage Plus Indirect Headset; Keeler). In addition, fluorescein staining was performed in both eyes of all animals following examination, tear testing, and tonometry. Horses with a history of ocular surface disease, concurrent health problems, or systemic antibiotic therapy within the 6 months before ocular surface sample collection were excluded from the study.

### 2.2 Sample size calculation

The sample size of 12 horses (6 per treatment group) was based upon a previously published equine fecal microbiota study in which an *a priori* power calculation determined that a sample size of 6 was necessary for a power of 0.80 with an alpha of 0.05 for detecting a 25% change in OTU counts, assuming a normal distribution with a mean ± SD OTU count of 2,886 ± 391/sample (Tyma et al., 2019). This sample size is also supported by the results of previous studies from our group in which sample sizes of 6–7 animals per group (with paired controls) were sufficient to yield significant differences in the relative abundance of the bacterial microbiota at all taxonomic levels (from phyla to genus level), and diversity indices (Costa et al., 2012, 2015).

### 2.3 Experimental design and sample collection

A prospective, randomized, blinded study design was utilized. Once confirmed to be free of ocular surface disease on examination, the horses were randomly assigned to one of two treatment groups (neomycin, polymyxin B and bacitracin [BNP] ophthalmic ointment [Bausch & Lomb] or neomycin, polymyxin B and dexamethasone [NPD] 0.1% ophthalmic ointment [Bausch & Lomb]) with a total of 6 horses in each group. These drug choices and dosing frequencies reflect standard treatment protocols commonly used for uncomplicated ocular surface and intraocular diseases in equine practice.

Depending on the group, one eye from each horse was randomly selected for treatment with the respective ophthalmic ointment, with the other eye being the control ([BNP-Co) and [NPD-Co]). Each horse received a ¼” strip of the respective ophthalmic ointment in the treatment eye and no treatment in the control eye three times daily for 7 days. Following day 7, medication administration was discontinued. Complete ophthalmic examinations, as described above, were repeated following discontinuation of medication (on day 9) and immediately before completion of the study (on day 30) to ensure no evidence of corneal ulceration or other ophthalmic changes developed secondary to medication administration or ocular surface sample collection.

Four to 6 h after treatment administration, one ocular surface sample was collected using rapid-drying sterile forensic swabs (GenoTube Livestock) run along the inferior conjunctival fornix (anterior to the third eyelid) of both eyes from all horses. Swabs were collected from the periocular skin of four selected treated eyes and collected at the different sampling points. The four horses were randomly selected for skin sampling using a manual draw where each horse’s ID was written on identical slips of paper, thoroughly mixed, and four slips were randomly drawn. A swab was held in the air for 30 s to provide environmental control at each time point. Samples were collected on Day 0 (following initial examination but before initiation of treatment), day 1, day 3, day 7, day 9, day 14, and day 30. The 30-day monitoring period was determined based on the results of a previously published microbiota study in horses, in which the pre-treatment fecal microbiota was reestablished by 25 days following the discontinuation of systemic antimicrobials (Gomez et al., 2023). The samples were packaged, labelled, and stored in an −80°C freezer until processing.

### 2.4 Sample sequencing and processing

The samples were shipped overnight to Argonne National Laboratory—Biosciences Division (BIO) Environmental Sample Preparation and Sequencing Facility (ESPSF) for processing (BIO Job No. 200207-1). DNA was extracted from all collected swabs. PCR amplification, library preparation and sequencing of the V4 region of the bacterial 16 s gene were performed at the Argonne Sequencing Center (Supplementary file 1).

### 2.5 Data analysis

The first 80 nucleotides were removed from the raw reverse reads to remove ambiguous nucleotides from Illumina reads. Afterwards, forward and reverse read lengths were truncated approximately when the average Phred score dropped below 30 (at 240 nt and 185 nt for forward and reverse reads, respectively). Sequence quality is presented in Supplementary Table 1. Amplicon sequence variants (ASVs) for forward and reverse reads were then inferred and merged with a minimum overlap of 50 bp using the DADA2 R package v. 1.14.1 (Callahan et al., 2016). Raw fastq reads generated from deep amplicon sequencing are accessible at the NCBI SRA database^1^ under BioProject accession number PRJNA1254175. Following chimera removal using the consensus method within DADA2, ASVs were taxonomically classified using the naïve Bayesian classifier in DADA2 against the SILVA v. 138.1 database (Quast et al., 2013). Finally, microbial taxa designated chloroplast or mitochondria were removed before downstream analysis.

All data analysis was performed in R v. 3.6.3. Before analysis, samples with less than 4,500 reads were discarded from downstream analysis. A read count of 4,500 was chosen to maintain the maximum number of samples possible with adequate sequencing depth. The remaining samples were rarefied to 4,500 reads for diversity analysis. Observed ASV richness and Shannon’s diversity estimates of alpha diversity were calculated. Linear mixed effect models were computed with the lme4 v. 1.1.23 R package to determine the association of day and treatment on alpha diversity metrics. Models were built with diversity index as the outcome, treatment group, day, and their interactions as fixed effects, while the horse was included as a random effect. Pairwise differences for model fixed effect variables were calculated using Tukey’s HSD in the emmeans v 1.6.0 R package. A *p*-value less than 0.05 was considered statistically significant.

Weighted and unweighted UniFrac distances were used to estimate phylogenetic differences in microbial composition between samples (Sfiligoi et al., 2022). For UniFrac distance measurements, a maximum likelihood tree was built with a general time reversible model against aligned DNA sequences of ASV using MUSCLE v. 3.8.31 and FastTree v. 2.1.11. Principal coordinate analysis (PCoA) and permutational multivariate analysis of variance (PERMANOVA) on UniFrac distances were used to associate treatment and time with microbial composition. PERMANOVAs were performed with 999 permutations stratified by horse, using treatment, day, and their interactions as predictors using the vegan 2.6.4 R package. Linear mixed-effect models were built using the same fixed and random effects as above to identify pairwise differences in microbial composition across treatment groups and day interactions. A *p*-value less than 0.05 was considered statistically significant.

Hierarchical clustering analysis was performed on samples from each time point to determine groups of samples similar in microbial composition. Ward’s minimum variance method was applied to weighted UniFrac distances for clustering. Differentially abundant bacterial families between the treatment group and day were determined using DESeq2 v. 1.26.0. DESeq2 formulas included horse (to account for paired samples) and treatment-day interactions. A p-value less than 0.01 was considered statistically significant for differential abundance analysis.

## 3 Results

An average of 30,158 ± 11,640 raw paired reads were obtained from Illumina sequencing. After sequence quality filtering and processing, 22,863 ± 8,769 reads were used for taxonomic classification. Few samples were removed from analysis due to low sequencing depth (BNP, n = 1; BNP-Co, *n* = 1; NPD-Co, *n* = 2; Supplementary Figure 1). The microbiota of skin and air swab control samples were analyzed before being removed from downstream analysis (Supplementary Figures 1–4). Air and skin swab samples had similar overall phyla distribution to ocular samples; however, air swabs had relatively low sequencing depths, and phyla relative abundance of skin samples appeared approximately constant over the day (Supplementary Figures 1A,B).

**Figure 1:**
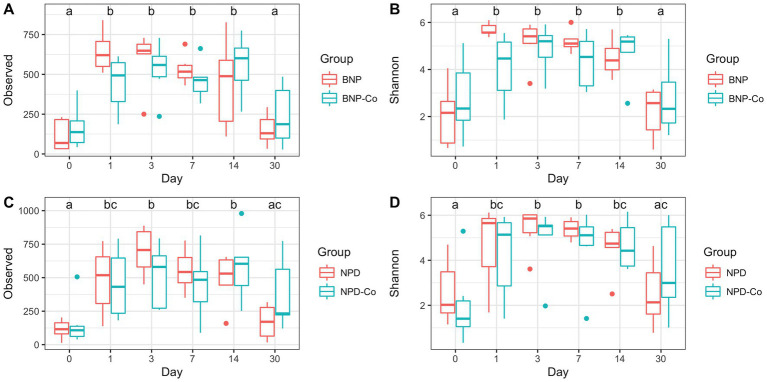
Alpha diversity boxplots of treatment groups by day. **(A)** Observed ASV counts for antibiotic and antibiotic-control groups. **(B)** Shannon index for antibiotic and antibiotic-control groups. **(C)** Observed ASV counts for antibiotic-corticosteroid and antibiotic-corticosteroid-control groups. **(D)** Shannon index for antibiotic-corticosteroid and antibiotic-corticosteroid-control groups. Different letters within a single panel represent a significant difference in alpha diversity metric over time. BNP, antibiotic (neomycin-polymyxin B-bacitracin ophthalmic ointment); BNP-Co, antibiotic-control; NPD, antibiotic-corticosteroid (neomycin-polymyxin B-dexamethasone 0.1% ophthalmic ointment); NPD-Co, antibiotic-corticosteroid-control.

A full characterization of ocular microbiota was completed for antibiotic-treated and antibiotic-corticosteroid-treated samples. Only day was statistically significant (*p* < 0.05) for observed richness and Shannon diversity in the linear mixed model. For all measurements, alpha diversity significantly increased (*p* < 0.05) from day 0 to day 1 before smoothing for the remainder of the sampled days, with a decrease on day 30 (Figures 1A–D).

**Figure 2:**
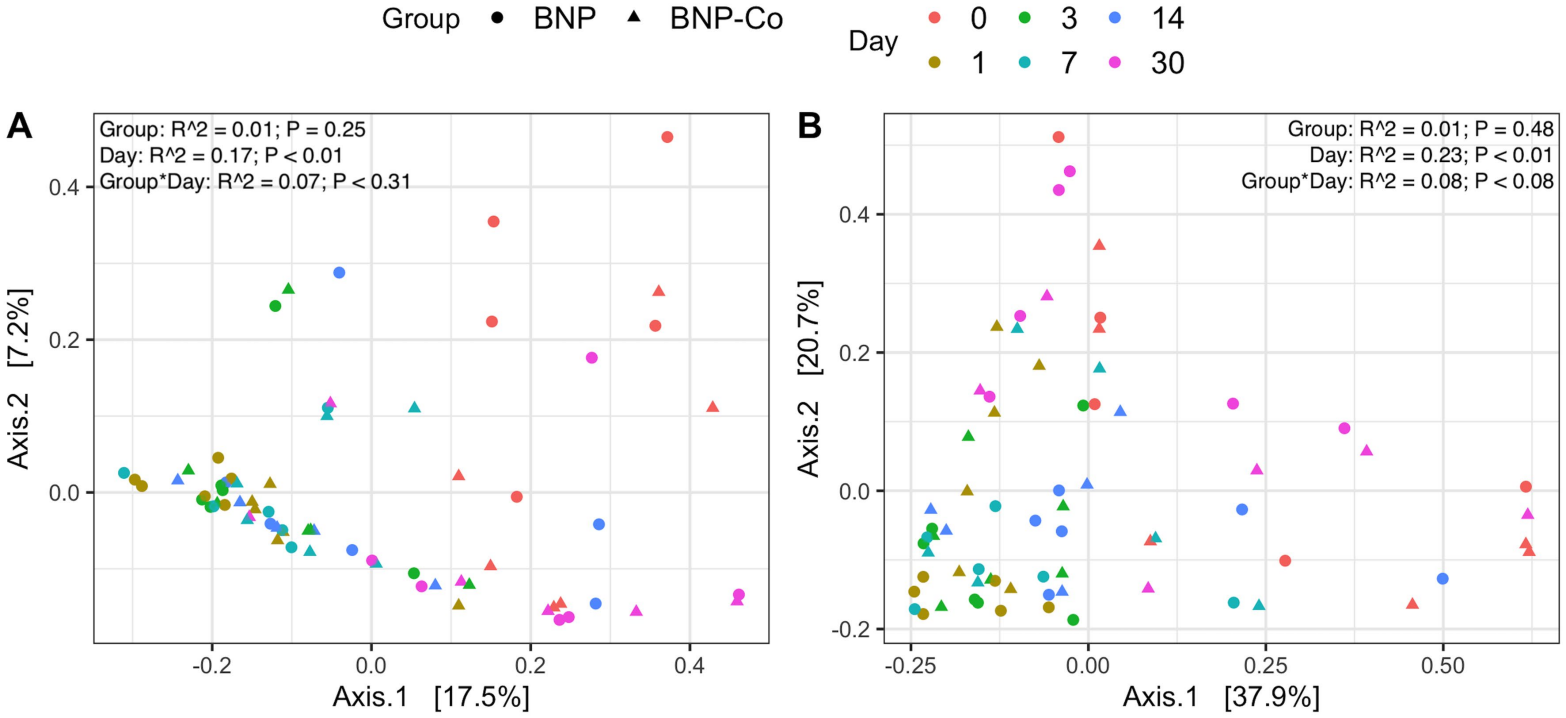
Principal coordinate analysis on UniFrac distances across antibiotic treatment and antibiotic-control samples. **(A)** Unweighted UniFrac distances. **(B)** Weighted UniFrac distances. Each color represents a different day, and the shape refers to whether samples were antibiotic-treated or antibiotic-control. BNP, antibiotic (neomycin-polymyxin B-bacitracin ophthalmic ointment); BNP-Co, antibiotic-control. PERMANOVA R^2^ and *p*-values are displayed for respective predictors in the upper right corner for each UniFrac metric.

**Figure 3:**
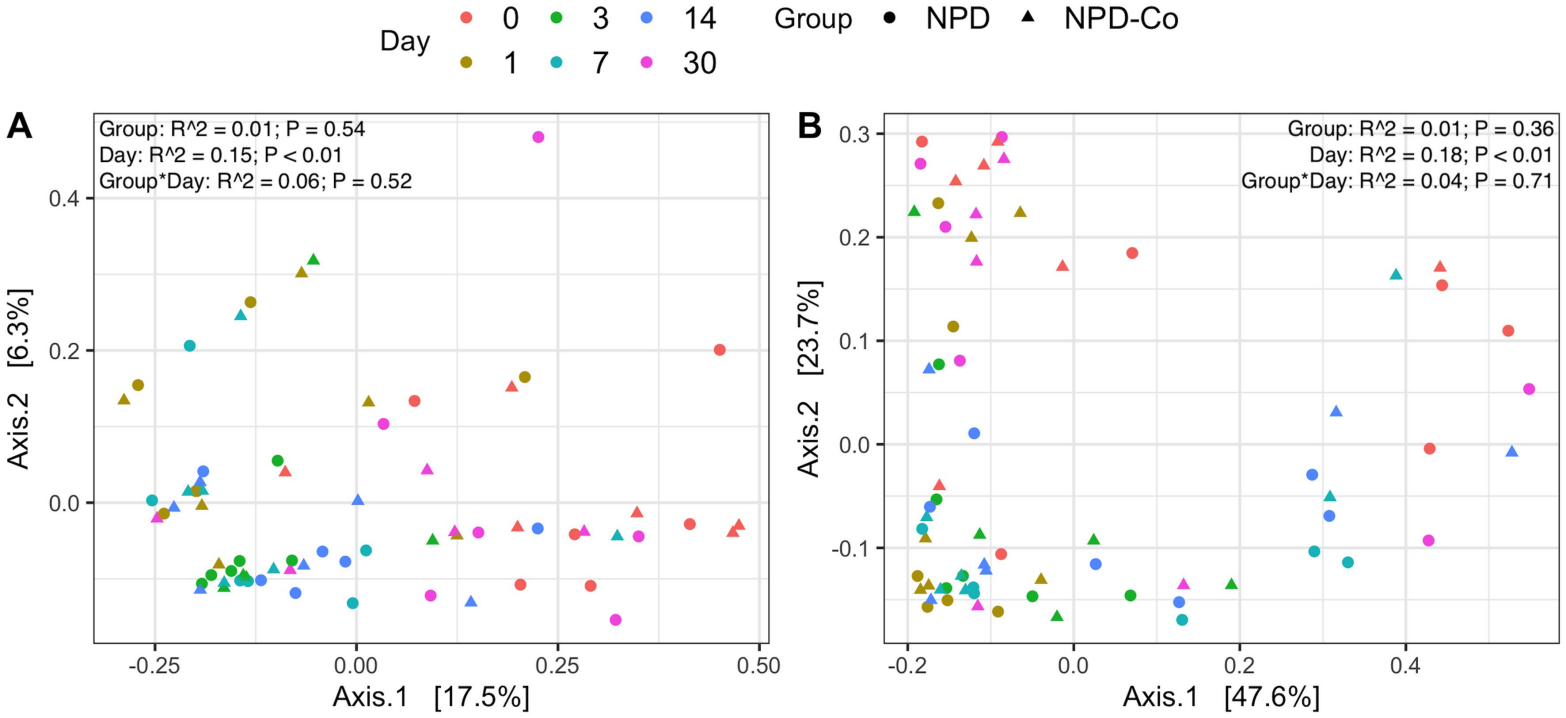
Principal coordinate analysis on UniFrac distances across antibiotic-corticosteroid treatment and antibiotic-corticosteroid-control samples. **(A)** Unweighted UniFrac distances. **(B)** Weighted UniFrac distances. Each color represents a different day, and the shape refers to if samples were from either the antibiotic-corticosteroid treated or antibiotic-corticosteroid control. NPD, antibiotic-corticosteroid (neomycin-polymyxin B-dexamethasone 0.1% ophthalmic ointment); NPD-Co, antibiotic-corticosteroid-control. PERMANOVA R^2^ and p-values are displayed for respective predictors in the upper right corner for each UniFrac metric.

**Figure 4:**
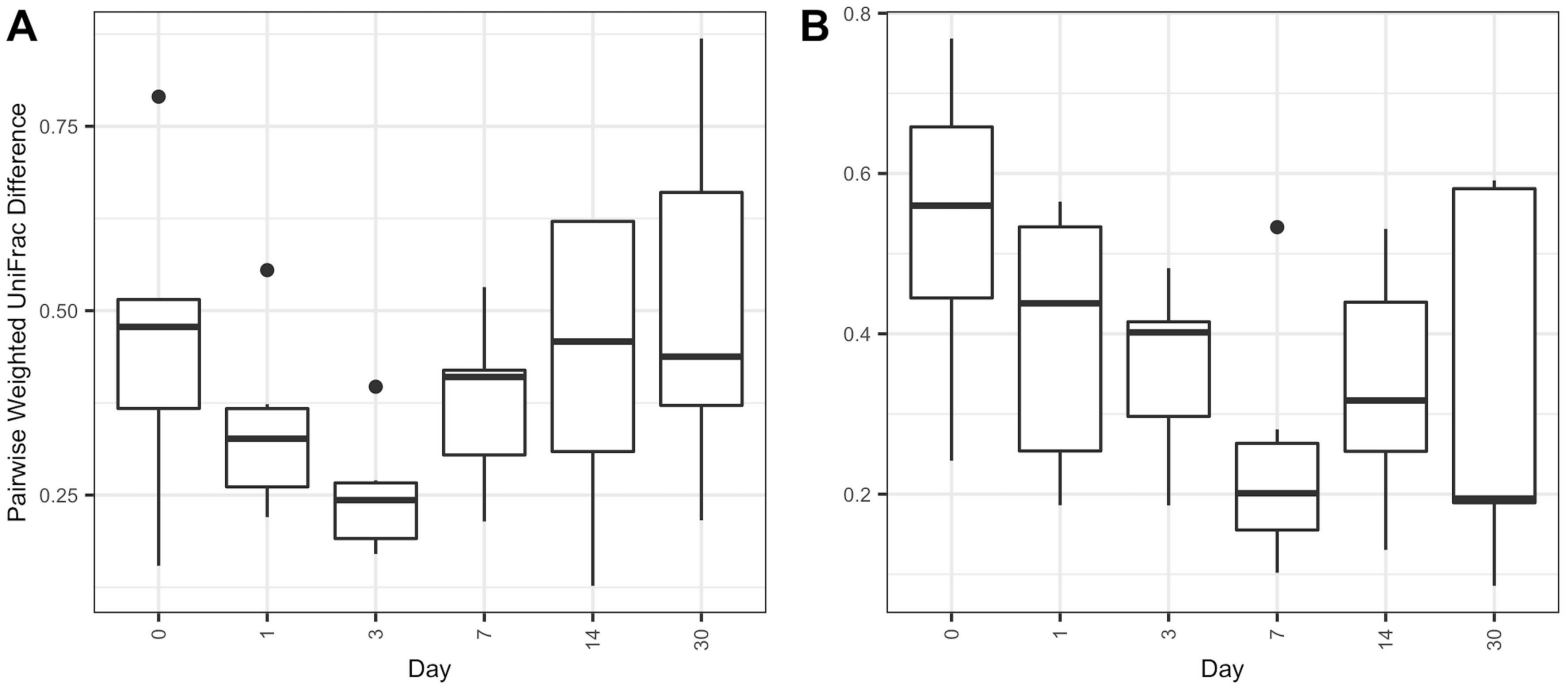
Boxplot of pairwise weighted UniFrac distances between treatment and control groups. Pairwise distances between treatments and controls were calculated within individual animals only. **(A)** Pairwise antibiotic and antibiotic-control weighted UniFrac distances. **(B)** Pairwise antibiotic-corticosteroid and antibiotic-corticosteroid-control weighted UniFrac distances.

Overall microbial composition of the ocular surface varied significantly (*p* < 0.05) across days for all distance metrics analyzed (Figures 2A,B, 3A,B). Neither treatment nor treatment-day interactions were statistically significant in PERMANOVA analyses. Principal coordinate analysis showed that weighted UniFrac distances exhibited a higher variation in microbial composition than unweighted UniFrac distances (Figures 2A,B, 3A,B). For BNP and BNP-Co samples, the principal coordinate analysis showed that day 1 to day 14 samples cluster together based on microbial composition, separately from day 0 and day 30 (Figure 2B). Similar clustering patterns were observed for NPD and NPD-Co samples (Figure 3B). Pairwise weighted UniFrac dissimilarity between BNP and BNP-Co samples show that these samples become increasingly similar until reaching the highest similarity at day 3 and return to day 0 levels after that (Figure 4A). NPD and NPD-Co have a similar pairwise dissimilarity, but samples were most similar in microbial composition at day 7 (Figure 4B). Hierarchical clustering analysis against weighted UniFrac distances showed that the relative abundance of Cardiobacteriaceae and Pasteurellaceae had proportionally large influences on the clustering of all samples based on microbial composition (Supplementary Figure 3).

### 3.1 Relative abundance

**Figure 5:**
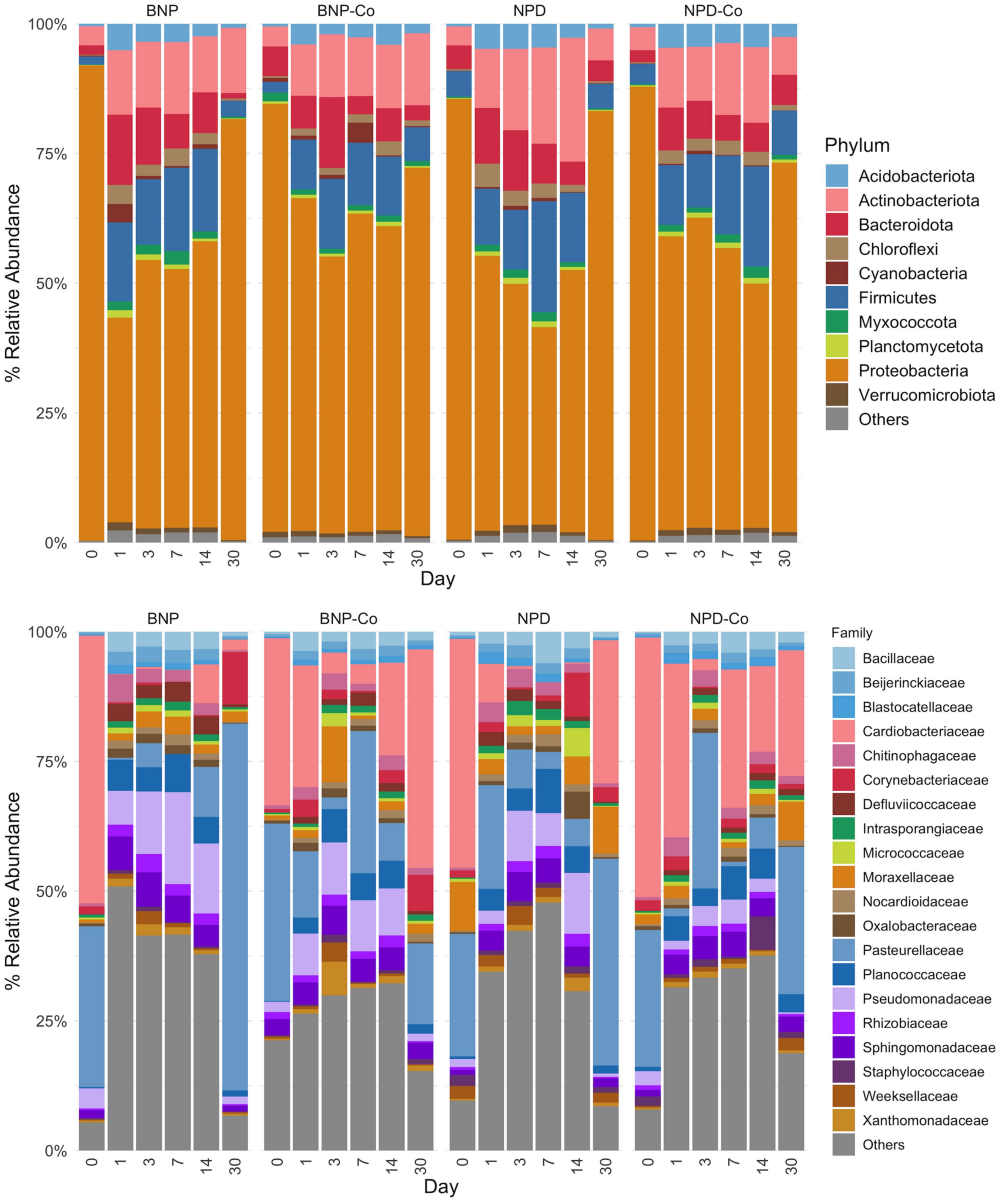
Relative abundance by day of ocular microbiota in treated and untreated eyes. The top 10 phyla and top 20 families by relative abundance metric are displayed, with the remaining taxa being grouped as “Others.” Samples were grouped according to treatment group. BNP, antibiotic (neomycin-polymyxin B-bacitracin ophthalmic ointment); BNP-Co, antibiotic-control; NPD, antibiotic-corticosteroid (neomycin-polymyxin B-dexamethasone 0.1% ophthalmic ointment); NPD-Co, antibiotic-corticosteroid-control.

Phylum-level distribution of ocular samples showed that Proteobacteria are the predominant phylum for all sample groups at day 0, present at greater than 75% relative abundance (Figure 5). By day 1 after treatment, the relative abundance of Proteobacteria declined substantially in all groups, but especially in BNP samples, to below 50% of the day 0 relative abundance (Figure 5). In contrast, Firmicutes abundance increased by day 1 after treatment in all groups (Figure 5). Bacteroidetes relative abundance increased on day 1 in the BNP group compared to the BNP-Co and remained higher throughout the study period until day 30, when it decreased to day 0 levels. Changes in Bacteroidetes were not evident in the NPD group. Actinobacteriota had a stepwise increase in relative abundance from day 0 to day 14 but decreased to day 0 levels by day 30 in all other groups (Figure 5).

**Figure 6:**
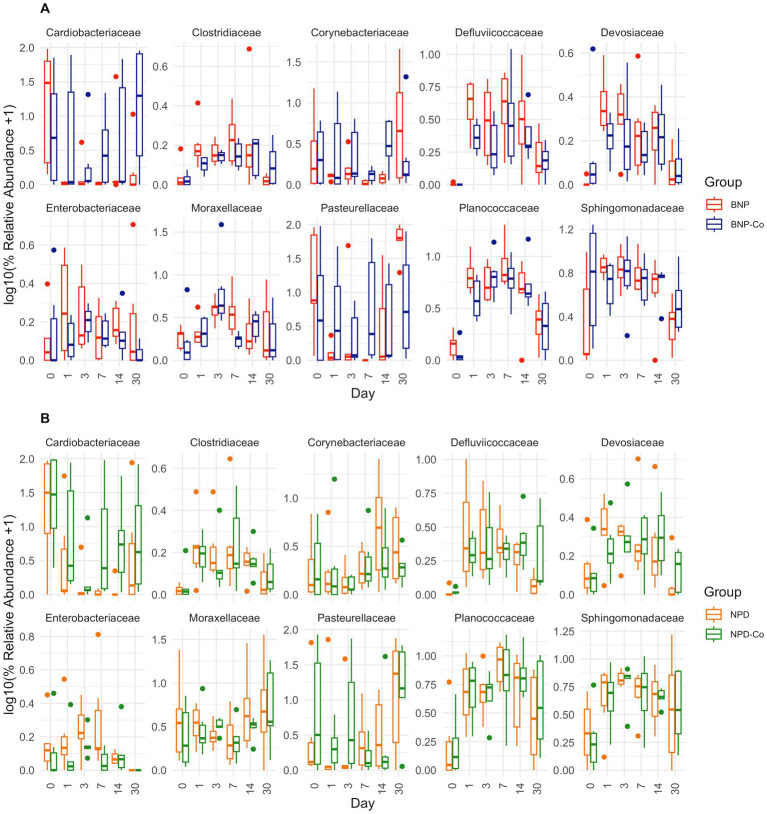
Relative abundance boxplots of ten bacterial families. Ten bacterial families were analyzed based on high relative abundance and statistical significance across days by DESeq2 differential abundance analysis. Boxplots are colored by treatment group, and relative abundance was transformed by log10 after a pseudocount of 1 was added. **(A)** BNP, antibiotic (neomycin-polymyxin B-bacitracin ophthalmic ointment); BNP-Co, antibiotic-control samples. **(B)** NPD, antibiotic-corticosteroid (neomycin-polymyxin B-dexamethasone 0.1% ophthalmic ointment); NPD-Co, antibiotic-corticosteroid-control samples. For p-values of statistically significant differences, refer to the text in the results section.

**Figure 7:**
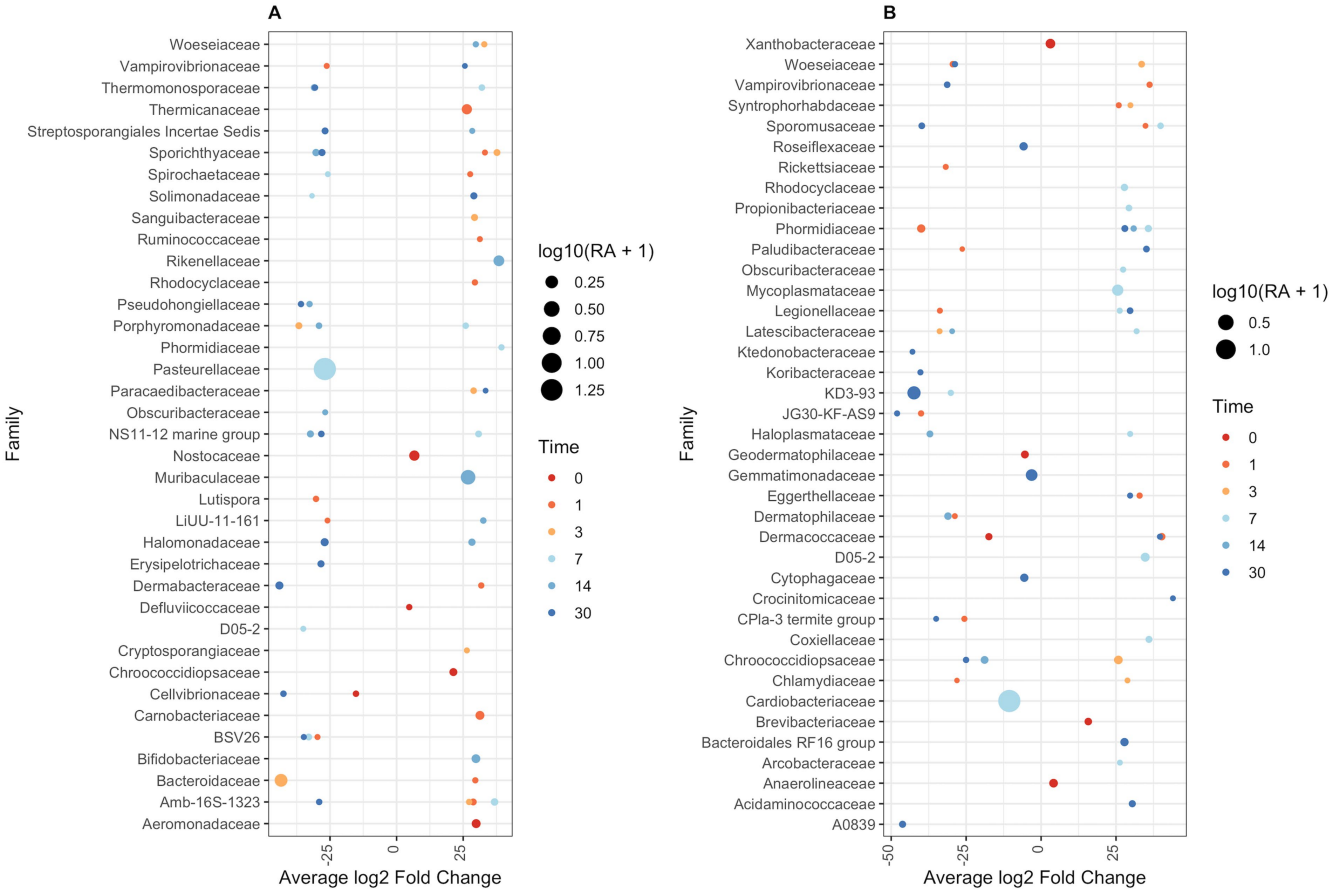
DESeq2 differential abundance analysis of bacterial families between paired treatment and control samples. Sequence read counts grouped at a family level were normalized using DESeq2. Normalized read counts were used to estimate fold change in bacterial family abundance between treatment and controls. **(A)** Antibiotic against controls. **(B)** Antibiotic-corticosteroid against controls. Only bacterial families at each time point that are significantly different in abundance across treatment control are shown (*p* < 0.01). Dots represent log2 fold-change of treatment bacterial family abundance against control abundance. Dot size is related to associated relative abundance (if fold-change is negative, then control group relative abundance is displayed; if fold-change is positive, then treatment group relative abundance is shown). Dots are coloured based on the day of sampling.

At family-level taxonomy, two families (i.e., Cardiobacteriaceae and Pasteurellaceae) comprised most of the day 0 microbiota (Figure 5). By day 1 in BNP samples, Cardiobacteriaceae and Pasteurellaceae significantly decreased (*p* < 0.01) and appeared almost absent from the ocular microbiota (Figure 6A). Pasteurellaceae significantly increases (*p* < 0.01) from day 14 to day 30 and becomes the predominant bacterial family in the BNP group microbiota, but Cardiobacteriaceae remained low in relative abundance throughout the study period (Figures 5, 6A). In contrast, Cardiobacteriaceae counts in BNP-Co samples appeared to decrease (*p* > 0.01) only on day 3 before returning to initial levels on day 7 (Figures 5, 6A). Pasteurellaceae counts in BNP-Co samples do not follow any significant (*p* > 0.01) trend throughout the time of measurement (Figures 5, 6A). Pasteurellaceae is significantly more abundant (*p* < 0.01) in BNP-Co samples than in BNP samples on day 7 (Figure 7A).

In NPD samples, Cardiobacteriaceae relative abundance significantly decreased (*p* < 0.01) on day 3 and remained low until day 30, when it significantly increased (*p* < 0.01). In contrast, in NPD-Co samples, Cardiobacteriaceae appears to decrease only on Day 3, but no statistically significant differences were observed (Figures 5, 6B). In addition, Cardiobacteriaceae counts were significantly higher in NPD samples compared to NPD-Co samples on day 7 (Figure 7B), and Pasteurellaceae showed a significant increase (*p* < 0.01) between days 14 and 30 for NPD samples compared to NPD-Co (Figures 5, 6B).

**Figure 8:**
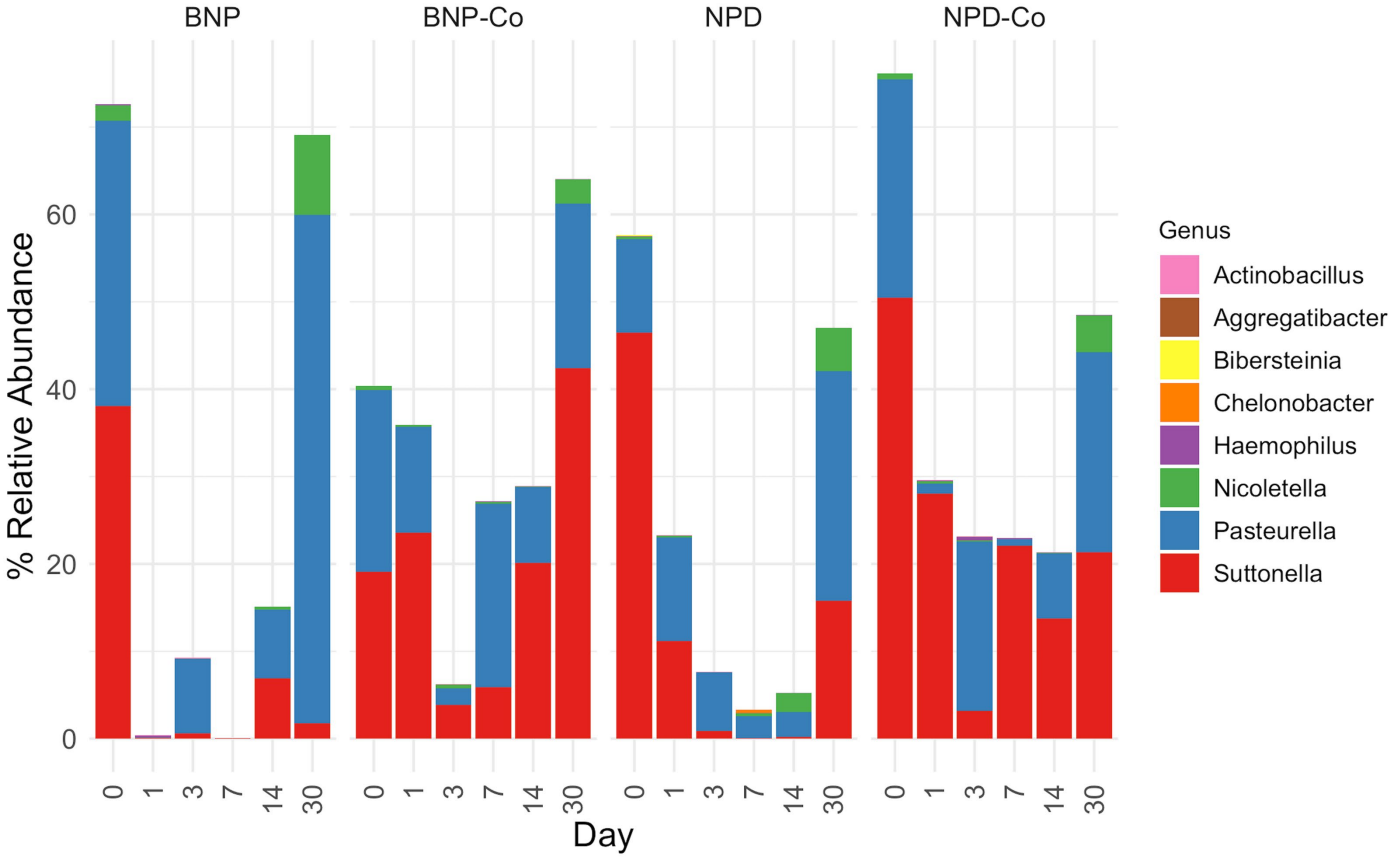
Relative abundance of genera from *Cardiobacteriaceae* and *Pasteurellaceae* families. Relative abundance was calculated before removing all genera outside the *Cardiobacteriaceae* and *Pasteurellaceae* families. Samples were grouped according to treatment group. BNP, antibiotic (neomycin-polymyxin B-bacitracin ophthalmic ointment); BNP-Co, antibiotic-control; NPD, antibiotic-corticosteroid (neomycin-polymyxin B-dexamethasone 0.1% ophthalmic ointment); NPD-Co, antibiotic-corticosteroid-control.

The primary genus-level taxa of Cardiobacteriaceae in all treatment groups and days measured was *Suttonella*; meanwhile, for Pasteurellaceae, it was *Nicoletella* and *Pasteurella* (Figure 8). *Nicoletella* relative abundance in all treatment groups was primarily associated with day 30 samples (Figure 8). The decrease in Cardiobacteriaceae and Pasteurellaceae family abundance during days 1 to 14 was associated with an increase in the relative abundance of multiple bacterial families (e.g., *Clostridiaceae, Defluviicoccaceae, Devosiaceae,* and *Planococcaceae* (Figures 5, 6A,B). Moraxellaceae family members, including *Moraxella*, were relatively stable in relative abundance throughout all days measured for all treatment groups (Figures 6A,B).

## 4 Discussion

The ocular surface microbiota of all horses in the study at baseline (day 0) was similar regardless of the treatment group. At the phylum level, Proteobacteria predominated at a relative abundance greater than 75% in all sample groups. Actinobacteriota, Bacteroidota, and Firmicutes were the next most observed phyla, each present in a much lower relative abundance in all sample groups. This finding is similar to previous studies evaluating the equine conjunctival microbiota in which Proteobacteria abundance varies between 41.6% (Scott et al., 2019) and 96.8% (LaFrentz et al., 2020) and Firmicutes between 0.9% (LaFrentz et al., 2020) to 24.6% (Scott et al., 2019). Similar trends in the most common phyla identified in the ocular surface are described in dogs and cats (Darden et al., 2019; Rogers et al., 2020).

At the family level in all sample groups, Cardiobacteriaceae and Pasteurellaceae predominated at day 0. The primary genus-level taxa of Cardiobacteriaceae was *Suttonella*, and the primary genus-level taxa of Pasteurellaceae were *Nicoletella* and *Pasteurella* in all treatment groups and days measured. Pasteurellaceae and the unclassified order of Cardiobacteriales are among the most common bacterial families identified on the ocular surface of horses, with relative abundances of 13.7 and 7.7%, respectively (Scott et al., 2019). Other commonly observed bacterial families were Sphingomonadaceae and Moraxellaceae, with relative abundances ranging from 4.8 and 7.9%, respectively (Scott et al., 2019). These bacteria have been reported in previous studies in healthy, untreated horses. In addition, *Nicolettela*, *Pasturella* and Moraxellacea are bacteria commonly detected in the nasal cavity of horses (Gomez et al., 2021). The nasolacrimal duct communicates the horses’ nasal cavity and ocular surface, which can also partially explain the similarities in the bacterial population identified in these body sites.

In the present study, gram-negative bacteria represented the most identified organisms on the equine ocular surface microbiota. This contrasts with the results of previous culture-based ocular surface studies in horses where the bacterial composition consisted primarily of gram-positive organisms such as *Streptococcus* spp. and *Staphylococcus* spp., *Bacillus* spp., *Corynebacterium* spp., and *Micrococcus* spp. (Whitley and Moore, 1984). The gram-negative bacteria *Pseudomonas aeruginosa* was also found in the culture of the ocular surface of healthy horses; however, it had a decreased incidence compared to gram-positive aerobes (Whitley and Moore, 1984). When characterized using high throughput techniques, this shift towards a gram-negative dominant ocular surface microbiota is documented in horses and other species, including humans, cats, and dogs (Darden et al., 2019; Leis and Costa, 2019; Scott et al., 2019; Rogers et al., 2020; Zhu et al., 2021), and likely reflects differences between both methodologies to characterize the microbiota.

Temporal changes were observed, with the overall microbial composition of the ocular surface varying significantly by sampling date. Many of these changes were similar or observed across all treatment groups. The changes in microbial communities were evident from day 1 and returned to baseline by day 30 with micro changes remaining. Contradictory results have been published regarding the effect of antibiotics on ocular surface microbiota, with studies showing a shift from predominantly gram-positive to gram-negative organisms when the microbiota is assessed using culture-based methodologies and others showing no alteration in the main taxa after antibiotic administration when the microbiota is characterized using high throughput sequencing methods (Whitley and Moore, 1984; Andrew et al., 1998; Gemensky-Metzler et al., 2005). In the antibiotic and antibiotic-corticosteroid treated eyes and their respective controls, a significant initial decrease in Proteobacteria with an increase in Firmicutes occurred. Cardiobacteriacea and Pasteureullaceae were the major family-level taxa that shifted over time, as they both comprised most of the ocular surface microbiota before treatments at day 0. Both families had significantly decreased relative abundance by day 1 in antibiotic-treated eyes and day 3 in antibiotic-corticosteroid eyes. Then, there was a recovery period during which Pastereullaceae increased considerably from day 14 to day 30 in the antibiotic and the antibiotic-steroid treated groups reaching baseline levels by day 30. These results differ from a previous study evaluating the effect of topical antibiotic ointment on the equine ocular surface microbiota, which found that the major bacterial taxa remained stable over time (Scott et al., 2019). The reasons for the disagreement between studies are unclear but could be explained by differences in sampling times, laboratory procedure and bioinformatic analysis. Of interest, studies assessing the impact of antibiotics on the fecal microbiota have reported that alterations in microbial communities start at day 1 after antibiotic administration, returning to baselines by day 30 (Liepman et al., 2022; Gomez et al., 2023).

The temporal changes observed in the ocular surface microbiota composition with an initial alteration from baseline after initiation of the topical therapy and a gradual return to the initial composition were observed in both treatment groups. This could indicate an effect of the ointment base and the medication on the local ocular surface environment through temporary alteration in variables such as tear film stability or tissue oxygen content. However, this appears less likely to be a contributing factor, as similar but delayed and slightly less pronounced changes were seen in the control groups. The ocular surface is an open system with constant environmental exposure and low biomass due to innate immune defense mechanisms (McDermott, 2013; Ozkan et al., 2017). The temporal variance in the ocular surface microbiota observed in this study may instead reflect a combination of the influence of environmental and husbandry conditions (e.g., running the horses into stocks and stables for ocular treatment and sampling) on the microbes present in the ocular surface. Also, local immune factors can be affected by medication administration and altered or upregulated in both treated and untreated eyes. Investigation on the individual effect of these factors on the ocular microbiota of horses is warranted.

There are several limitations to the current study. First, the sample size of 12 horses, with 6 in each treatment group, was determined based on a previous microbiota study; however, this study evaluated the gastrointestinal microbiota (Tyma et al., 2019). The gastrointestinal microbiota is a much higher biomass site than the ocular surface microbiota (Ozkan et al., 2018). It is possible that our sample size was small to detect differences accurately. With a smaller sample size, the study may be more susceptible to outlier animals with abnormally high or low relative abundance taxa when compared to the rest of the group. Additionally, when interpreting the results of microbiota studies and analyzing relative abundance plots, there may be visual differences that are not statistically significant or statistically significant but not clinically relevant. Future studies evaluating the stability of the ocular surface microbiota over a more extended period or seasonally with a larger number of horses would help increase the study’s power and better differentiate the impact of those factors in the equine ocular surface microbiota.

Additional limitations exist when considering the analysis of results from microbiota studies using next-generation sequencing techniques. There is a sequencing limitation in that 16S amplicons become less reliable in taxonomic classification as taxonomic resolution increases. Relative abundance of predominant taxa is only part of the study; it does not provide any information on the absolute quantity of each organism present. The changes in relative abundance cannot be distinguished if an increase in relative abundance is due to the increase in the actual bacterial count or a decrease in the amount of other bacterial groups present. This is a significant limitation of high-throughput sequencing when studying microbial populations longitudinally. It can be addressed using quantitative polymerase chain reaction (qPCR) of identified organisms to determine the actual bacterial load (Banks et al., 2019). Caution should also be taken when comparing the results of different high throughput studies due to many possible variations in study design regarding specific DNA extraction, sequencing, and analysis techniques. Moving forward with future studies using high-throughput sequencing, attention in study planning should be directed to normalize the workflow and ensure the consistent use of controls, improving reproducibility and allowing for more appropriate interpretation of results and comparison between studies (Banks et al., 2019; Scott et al., 2021). Finally, our analysis focused primarily on highly abundant species. However, recent research on low-abundance microbes within microbial communities highlights that their limited presence does not equate to minimal importance. These studies demonstrate that certain rare taxa can have a significant influence on community structure and host-related functions despite their low abundance (Jia et al., 2010; Han and Vaishnava, 2023). Gaining insight into the functions of low-abundance microbes is essential for uncovering the full complexity of host–microbe interactions. Nonetheless, our study showed that administering topical antibiotics with or without corticosteroids induces changes in the ocular surface of the horses’ eyes and that the microbial communities return to baseline characteristics approximately 3 weeks after cessation of treatment. Further research involving horses affected by ocular disease is warranted to determine changes in the ocular microbiota during disease progression and recovery and help guide more targeted and effective treatment strategies.

## Data Availability

The datasets presented in this study can be found in online repositories. The names of the repository/repositories and accession number(s) can be found at: https://figshare.com/, 10.6084/m9.figshare.27909534.

## Footnotes

^1^ www.ncbi.nlm.nih.gov/sra

## Supplementary material

SUPPLEMENTARY FIGURE 1 Sequence count and bacterial phyla distribution by sample type. **(A)** Boxplot of log_10_ transformed sequence counts for each sample type by day. **(B)** Relative abundance of each sample type by day. Bacteria were grouped at phylum level taxonomy, and phyla with less than 2% mean relative abundance were grouped as “other”. BNP, antibiotic (neomycin-polymyxin B-bacitracin ophthalmic ointment); BNP-Co, antibiotic-control; NPD, antibiotic-corticosteroid (neomycin-polymyxin B-dexamethasone 0.1% ophthalmic ointment); NPD-Co, antibiotic-corticosteroid-control; BNPSk, antibiotic-treated periocoular skin sample; NPDSk, antibiotic-corticosteroid-treated periocular skin sample.

SUPPLEMENTARY FIGURE 2 Alpha diversity for skin samples by day. **(A)** Observed ASV count. **(B)** Shannon index. Each line represents a unique sample, colored by the corresponding treatment applied to the sampled animal eye (antibiotic or antibiotic-corticosteroid). BNPSk, antibiotic-treated periocular skin sample; NPDSk, antibiotic-corticosteroid-treated animal skin sample. **(C)** InvSimpson. **(D)** Fisher.

SUPPLEMENTARY FIGURE 3 Principal coordinate analysis on UniFrac distances across skin samples. **(A)** Unweighted UniFrac distances. **(B)** Weighted UniFrac distances. Each color represents a different day, and the shape refers to the treatment applied to the sampled animal eye (antibiotic or dexamethasone). PERMANOVA R^2^ and p-values are displayed for respective predictors in the upper right corner for each UniFrac metric. BNPSk, antibiotic-treated periocular skin sample; NPDSk, antibiotic-corticosteroid-treated periocular skin sample.

SUPPLEMENTARY FIGURE 4 Heatmap of ocular microbiota distribution across treatment group and day. The top 50 abundant bacterial families are shown for clarity. Dendrograms on the sample group were created with hierarchical clustering using Ward’s method on weighted UniFrac distances. Samples are labelled as treatment groups concatenated with a day of sampling. BNP, antibiotic (neomycin-polymyxin B-bacitracin ophthalmic ointment); BNP-Co, antibiotic-control; NPD, antibiotic-corticosteroid (neomycin-polymyxin B-dexamethasone 0.1% ophthalmic ointment); NPD-Co, antibiotic-corticosteroid-control.

SUPPLEMENTARY FILE S1 Protocol for 16S rRNA amplicon library preparation and sequencing.
