# Supplementary figures and images for: The effect of topical antibiotic or antibiotic-corticosteroid treatment on the ocular surface microbiota of healthy horses
Source: Front Microbiol. 2025 Aug 4;16:1535095. doi: 10.3389/fmicb.2025.1535095 (PMC12359475; doi:10.3389/fmicb.2025.1535095)

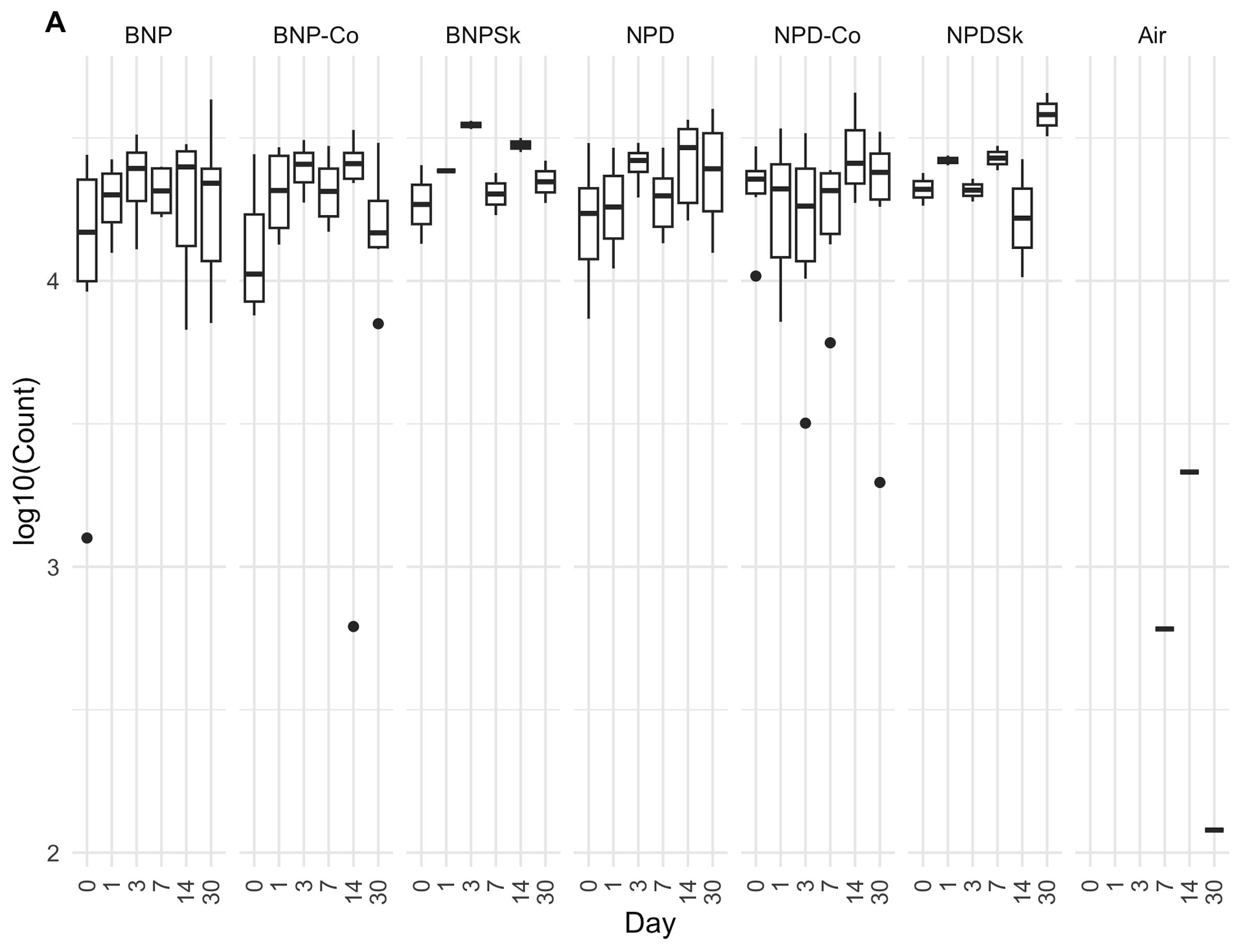

Supplement: SUPPLEMENTARY FIGURE 1 — Sequence count and bacterial phyla distribution by sample type. (A) Boxplot of log10 transformed sequence counts for each sample type by day. (B) Relative abundance of each sample type by day. Bacteria were grouped at phylum level taxonomy, and phyla with less than 2% mean relative abundance were grouped as “other”. BNP, antibiotic (neomycin-polymyxin B-bacitracin ophthalmic ointment); BNP-Co, antibiotic-control; NPD, antibiotic-corticosteroid (neomycin-polymyxin B-dexamethasone 0.1% ophthalmic ointment); NPD-Co, antibiotic-corticosteroid-control; BNPSk, antibiotic-treated periocoular skin sample; NPDSk, antibiotic-corticosteroid-treated periocular skin sample. [file Image_1.tiff]

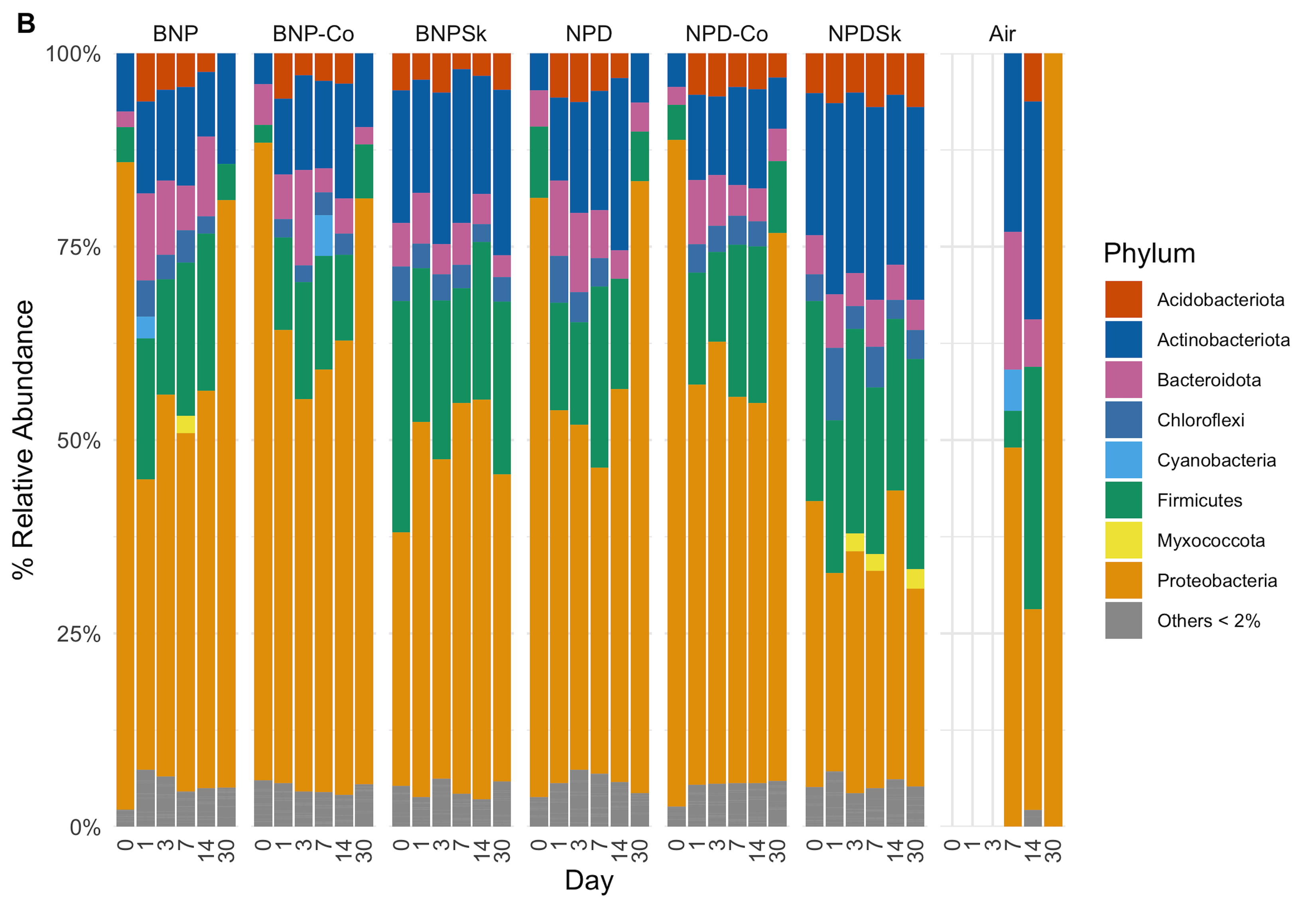

Supplement: SUPPLEMENTARY FIGURE 2 — Alpha diversity for skin samples by day. (A) Observed ASV count. (B) Shannon index. Each line represents a unique sample, colored by the corresponding treatment applied to the sampled animal eye (antibiotic or antibiotic-corticosteroid). BNPSk, antibiotic-treated periocular skin sample; NPDSk, antibiotic-corticosteroid-treated animal skin sample. (C) InvSimpson. (D) Fisher. [file Image_2.tiff]

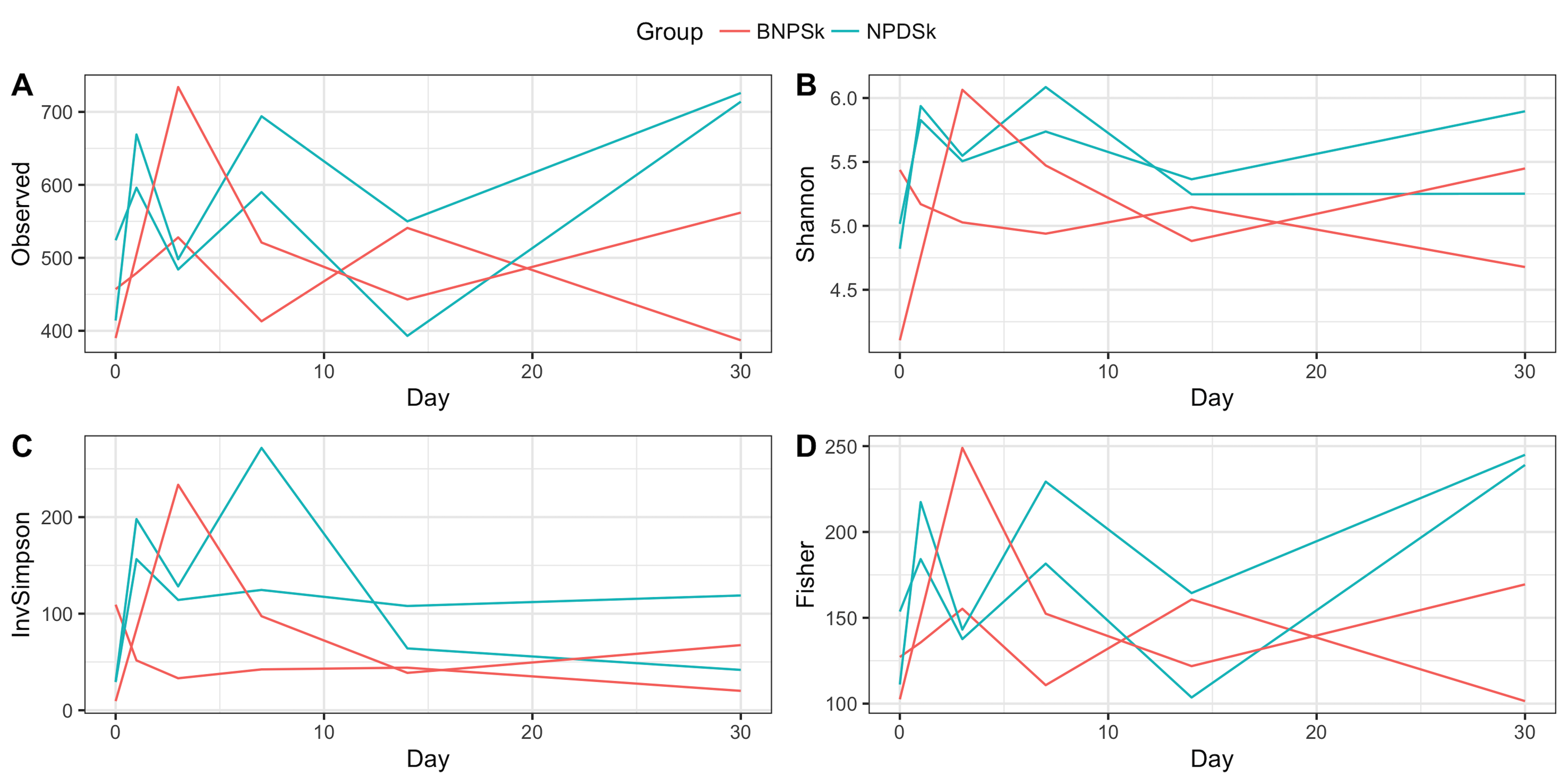

Supplement: SUPPLEMENTARY FIGURE 3 — Principal coordinate analysis on UniFrac distances across skin samples. (A) Unweighted UniFrac distances. (B) Weighted UniFrac distances. Each color represents a different day, and the shape refers to the treatment applied to the sampled animal eye (antibiotic or dexamethasone). PERMANOVA R2 and p-values are displayed for respective predictors in the upper right corner for each UniFrac metric. BNPSk, antibiotic-treated periocular skin sample; NPDSk, antibiotic-corticosteroid-treated periocular skin sample. [file Image_3.tiff]

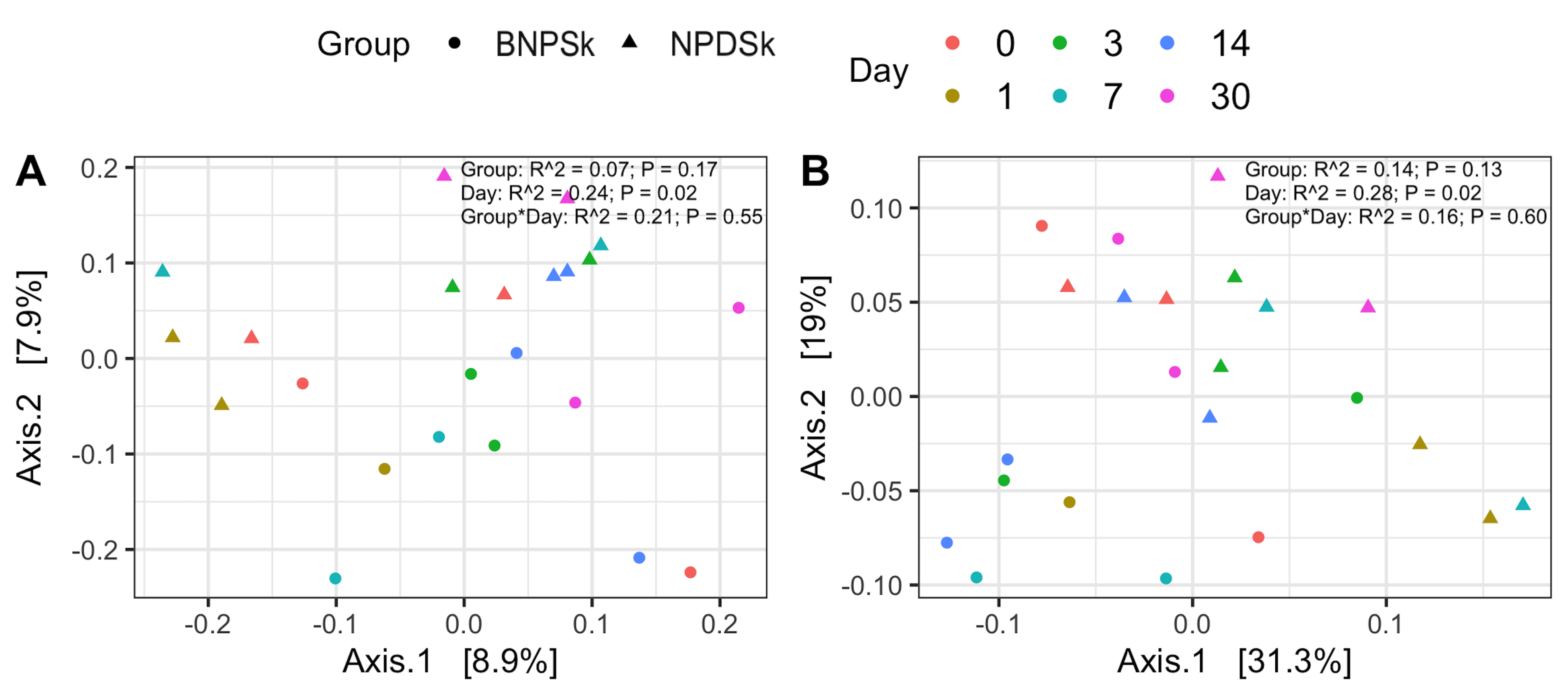

Supplement: SUPPLEMENTARY FIGURE 4 — Heatmap of ocular microbiota distribution across treatment group and day. The top 50 abundant bacterial families are shown for clarity. Dendrograms on the sample group were created with hierarchical clustering using Ward’s method on weighted UniFrac distances. Samples are labelled as treatment groups concatenated with a day of sampling. BNP, antibiotic (neomycin-polymyxin B-bacitracin ophthalmic ointment); BNP-Co, antibiotic-control; NPD, antibiotic-corticosteroid (neomycin-polymyxin B-dexamethasone 0.1% ophthalmic ointment); NPD-Co, antibiotic-corticosteroid-control. [file Image_4.tiff]

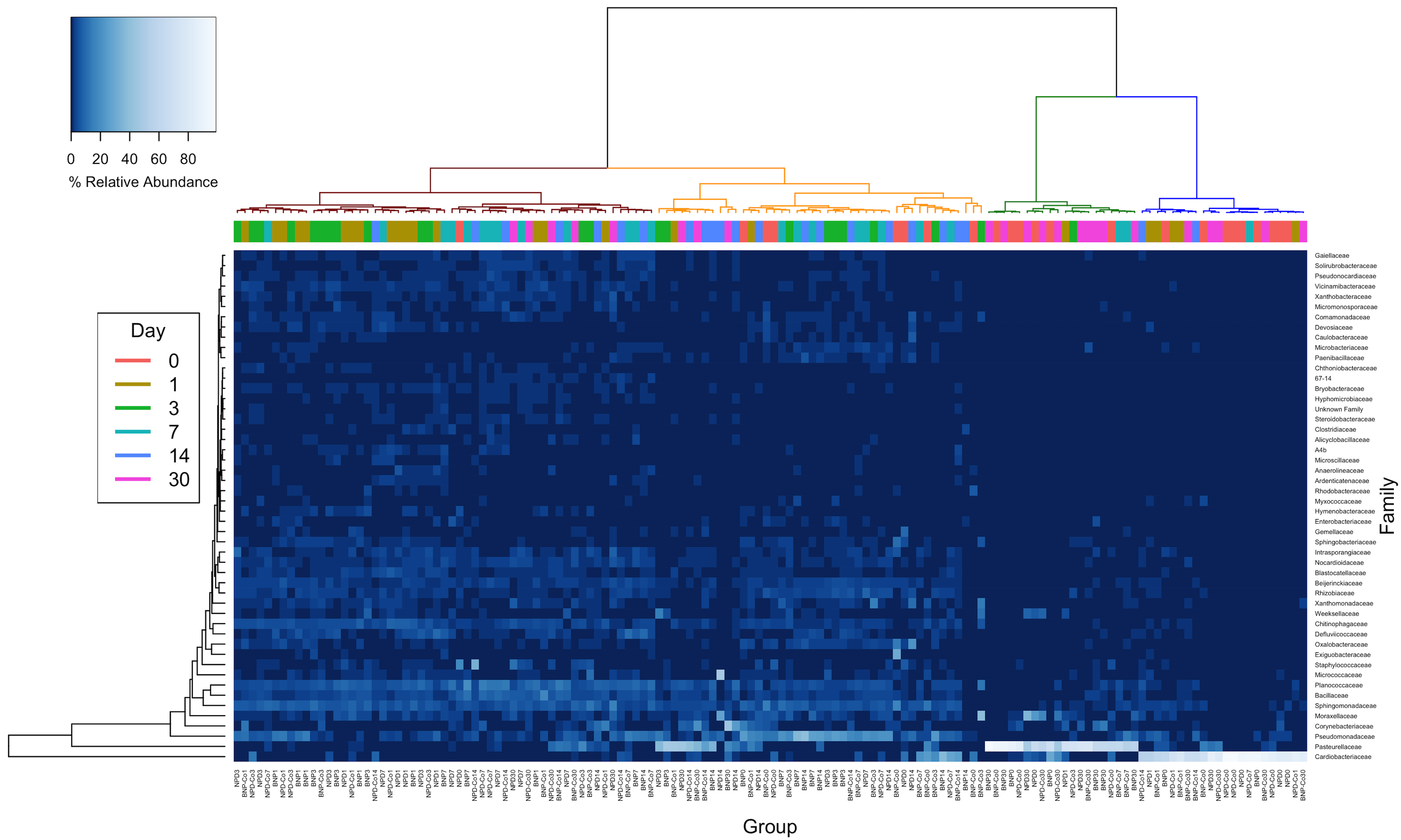

Supplement: SUPPLEMENTARY FILE S1 — Protocol for 16S rRNA amplicon library preparation and sequencing. [file Image_5.tiff]
