# Supplementary material for: The effect of topical antibiotic or antibiotic-corticosteroid treatment on the ocular surface microbiota of healthy horses
Source: Front Microbiol. 2025 Aug 4;16:1535095. doi: 10.3389/fmicb.2025.1535095 (PMC12359475; doi:10.3389/fmicb.2025.1535095)
Supplement: Supplementary file 6 [file Table_1.docx]

**Protocol for 16S rRNA amplicon library preparation and sequencing**

Briefly, PCR amplicon libraries targeting the 16S rRNA encoding gene present in metagenomic DNA are produced using a barcoded primer set adapted for the Illumina HiSeq2000 and MiSeq (1). DNA sequence data is then generated using Illumina paired-end sequencing at the Environmental Sample Preparation and Sequencing Facility (ESPSF) at Argonne National Laboratory. Specifically, the V4 region of the 16S rRNA gene (515F-806R) is PCR amplified with region-specific primers that include sequencer adapter sequences used in the Illumina flowcell (1, 2, 3, 4, 5).  The forward amplification primer also contains a twelve base barcode sequence that supports pooling of up to 2,167 different samples in each lane (1,2,3,4,5).

Primers and primer constructs were designed by Greg Caporaso (2011, 2012). Modifications to primer degeneracy were done by the labs of Jed Furhman (Parada et al., 2016) and Amy Apprill (Apprill et al., 2015). Forward-barcoded constructs were redesigned by Walters et al. (2016) based upon the original constructs generated by Caporaso et al. (2012).

Each 25 µL PCR reaction contains 9.5 µL of MO BIO PCR Water (Certified DNA-Free), 12.5 µL of QuantaBio’s AccuStart II PCR ToughMix (2x concentration, 1x final), 1 µL Golay barcode tagged Forward Primer (5 µM concentration, 200 pM final), 1 µL Reverse Primer (5 µM concentration, 200 pM final), and 1 µL of template DNA. The conditions for PCR are as follows: 94 °C for 3 minutes to denature the DNA, with 35 cycles at 94 °C for 45 s, 50 °C for 60 s, and 72 °C for 90 s; with a final extension of 10 min at 72 °C to ensure complete amplification. Amplicons are then quantified using PicoGreen (Invitrogen) and a plate reader (Infinite® 200 PRO, Tecan). Once quantified, volumes of each of the products are pooled into a single tube so that each amplicon is represented in equimolar amounts. This pool is then cleaned up using AMPure XP Beads (Beckman Coulter), and then quantified using a fluorometer (Qubit, Invitrogen). After quantification, the molarity of the pool is determined and diluted down to 2 nM, denatured, and then diluted to a final concentration of 6.75 pM with a 10% PhiX spike for sequencing on the Illumina MiSeq. Amplicons are sequenced on a 251bp x 12bp x 251bp MiSeq run using customized sequencing primers and procedures (1).

**References**

1. Caporaso JG*, et al.* (2012) Ultra-high-throughput microbial community analysis on the Illumina HiSeq and MiSeq platforms. *The ISME journal* 6(8):1621-1624.

2. Caporaso JG*, et al.* (2011) Global patterns of 16S rRNA diversity at a depth of millions of sequences per sample. *Proc Natl Acad Sci U S A* 108 Suppl 1:4516-4522.

3. Walters, *et al.* (2016) Improved Bacterial 16S rRNA Gene (V4 and V4-5) and Fungal Internal Transcribed Spacer Marker Gene Primers for Microbial Community Surveys. mSystems 1(1):e00009-15.

4. Apprill, *et al.* (2015) Minor revision to V4 region SSU rRNA 806R gene primer greatly increases detection of SAR11 bacterioplankton. Aquatic Microbial Ecology, 75(2), 129–137.

5. Parada, *et al.* (2016) Every base matters: assessing small subunit rRNA primers for marine microbiomes with mock communities, time series and global field samples. Environmental Microbiology, 18(5), 1403–1414.
